# Serum lipid levels in relation to clinical outcomes in pregnant women with gestational diabetes mellitus: an observational cohort study

**DOI:** 10.1186/s12944-021-01565-y

**Published:** 2021-09-29

**Authors:** Yuan Li, Xiaoqian Wang, Fengjuan Jiang, Wenqing Chen, Jie Li, Xiaotian Chen

**Affiliations:** 1 Department of Clinical Nutrition, Nanjing Drum Tower Hospital, the Affiliated Hospital of Nanjing University Medical School, Nanjing, Jiangsu 210008 China; 2 Department of Gynaecology and Obstetrics, Nanjing Drum Tower Hospital, the Affiliated Hospital of Nanjing University Medical School, Nanjing, Jiangsu 210008 China

**Keywords:** Lipids, Pregnancy, Gestational diabetes mellitus, Clinical outcomes

## Abstract

**Background:**

Research on dyslipidemia during pregnancy in women with gestational diabetes mellitus (GDM) has rarely been conducted in Asia. The present study aimed to evaluate maternal mid-trimester lipid profile in relation to GDM and clinical outcomes in these high-risk populations.

**Methods:**

The medical records of 632 pregnant women in the second trimester were retrospectively analyzed. Maternal fasting serum lipids were assayed for total cholesterol (TC), triglycerides (TG), high-density lipoprotein-cholesterol (HDL-C), low-density lipoprotein-cholesterol (LDL-C), apolipoprotein A1 (Apo A1) and Apo B concentrations during the second trimester. The atherogenic index of plasma (AIP) was calculated as log (TG/HDL). The clinical outcomes were collected by evaluating delivery mode, postpartum hemorrhage, prematurity, macrosomia, birth weight, body length and neonatal Apgar 5 min score.

**Results:**

Levels of TG and AIP were elevated while decreased HDL-C was observed in women with GDM compared with that of the control group. Significant differences were observed in gestational weeks at birth, cesarean section, postpartum hemorrhage, birth weight, body length, prematurity and macrosomia between the two groups. Compared with women with hyperlipidemia, the incidence of GDM and cesarean section was lower in normal lipid group. Women in the hyperlipidemia group had smaller gestational weeks at birth than those in the control group. According to the logistic regression analysis, each unit elevation in AIP increased the risk of GDM by 18.48 times (OR = 18.48, CI: 2.38–143.22). Besides, age (OR = 1.11, CI: 1.06–1.16) and pre-pregnancy BMI (OR = 1.15, CI: 1.07–1.24) were the risk factors of GDM.

**Conclusions:**

These findings suggested that reasonable lipid control in the second trimester might reduce the incidence of GDM and be a potential strategy for improving clinical outcomes in these high-risk women.

## Background

Maternal energy metabolism in the second half of pregnancy is directed toward lipolysis, which indicates a physiological adaptation of mothers to maintain stable fuel supplementation to the fetus [1, 2]. Mildly increased lipidemia occurs in early pregnancy, with a more pronounced elevation by the second and third trimesters [2–4]. However, it is still difficult to ascertain which level of lipid elevation is physiological or pathological nowadays. There is no consensus on which criteria should be used in diagnosis of hyperlipidemia during gestation in China.

The body suffers a substantial increase in the workload of different organs during pregnancy, which may be more likely to result in metabolic diseases. Gestational diabetes mellitus (GDM) is a common metabolic complication in pregnant women and affects up to 22% of pregnancies [5]. Pregnant persons with GDM are at increased risk for maternal and neonatal complications, including cesarean delivery, postpartum hemorrhage, preterm birth, macrosomia, small for gestational age (SGA) and low Apgar score [6–8].

Dyslipidemia is a well recognized risk factor for metabolic syndromes. Earlier studies have reported an increase lipid profile in patients with GDM compared to women without GDM [9]. Evidence also suggests that hypertriglyceridemia is one of the most prevalent modifications in those pregnancies complicated with GDM [5]. Nevertheless, the role of lipids profiles in predicting GDM is a controversial issue [10, 11]. Maternal dyslipidemia not only leads to pregnancy complications but also adverse perinatal outcomes [1]. Mudd et al. reported that elevated levels of total cholesterol (TC), low-density lipoprotein-cholesterol (LDL-C) and triglycerides (TG) at 15–27 weeks of gestation were linked to an increased risk of spontaneous preterm delivery [12]. Recent studies described a positive association between gestational dyslipidaemia and the adverse birthweight outcomes [13]. Additionally, pregnancy-induced hyperlipidemia has also been proved to be closely linked with cesarean delivery and postpartum hemorrhage [14]. Though moderately increased lipidemia preforms a fundamental role during pregnancy, there are still controversies on the relationship between maternal lipid disturbances and perinatal outcomes [15].

Although changes in lipid profile are expected, there is a shortage of consensus regarding reference values per trimester and the diagnostic criteria for hyperlipidemia. In addition, the use of lipid-lowering drugs during pregnancy is relatively contraindicated. Thus, evaluating the diagnostic criteria of hyperlipidemia and its association with adverse pregnancy outcomes is urgently needed, which may allow for appropriate and timely intervention. In the present study, we examined the blood lipids in patients with GDM, and analyzed putative relations with lipid profiles and clinical outcomes.

## Methods

### Study population

**Fig. 1:**
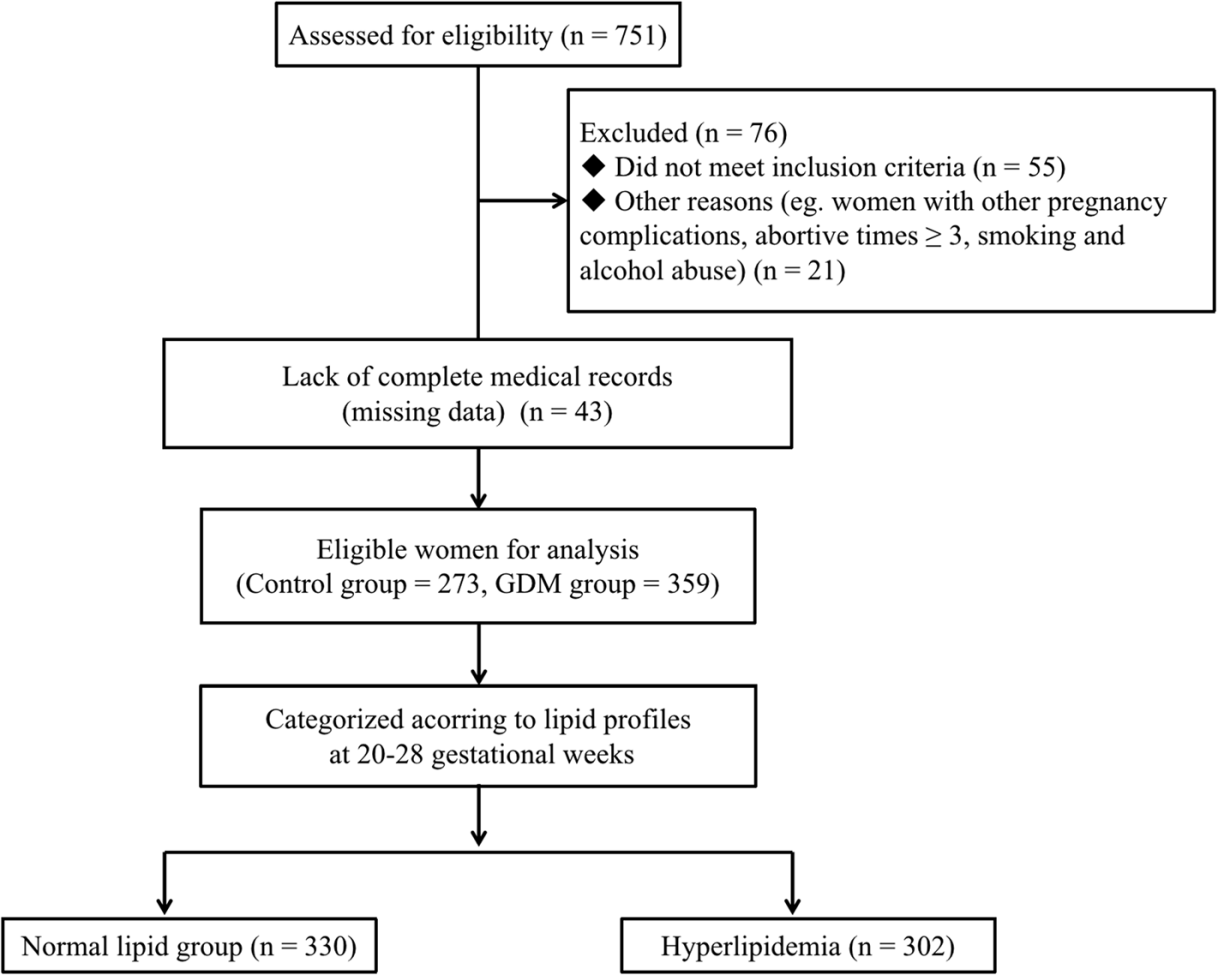
Flowchart of participants included in this study

Pregnant women who were admitted to Nanjing Drum Tower Hospital and delivered between January 2015 and December 2017 were recruited for the present study. The inclusion criteria were as follows: (1) age between 20 to 40 years, (2) a lipid measurement at 20–28 gestational weeks, (3) regularly for prenatal examination in the Drum Tower Hospital; We excluded: (1) Incomplete data of medical records; (2) women with other pregnancy complications, medical or surgical diseases (i.e a specific psychiatric disorder, hypertensive disorders, immunological, liver and kidney diseases); (3) abortive times ≥3, (4) twin or multiple pregnancy, (5) received assisted reproductive technology, (6) the use of insulin therapy or hypoglycaemic drugs, (7) previous diagnosis of gestational diabetes (or diabetes) and (8) smoking, alcohol abuse and long-term medication treatment (i.e anti-hyperglycemic and anti-hyperlipidemic drugs). Considering a type I error of 5% (α = 0.05), a study power of 80% and the incidence of GDM as key variable, we estimated a sample size of sixty persons for each group at least. Finally, 632 pregnant women (*n* = 273 in control group, *n* = 359 in GDM group) were enrolled in the final analysis. Figure 1 shows the participant flow.

### Data collection

Data were extracted from medical records. General characteristics of the study population included maternal age, gestational age at blood collection, pre-pregnancy body mass index (BMI), gravidity, parity, permanent residence (urban or rural areas), monthly income and education attainment. Maternal metabolic parameters included serum TG, TC, HDL-C, LDL-C Apo A1 and Apo B. The atherogenic index of plasma (AIP) = log (TG/HDL). Other clinical data were collected by evaluating delivery mode, postpartum hemorrhage, prematurity, macrosomia, birth weight, body length and neonatal Apgar 5 min score. Preterm birth refers to as birth of newborns less than 37 weeks gestational age. Macrosomia is defined as a fetal birth weight equal to or greater than 4000 g.

Pregnant women should subject to GDM screening universally at 24–28 weeks of gestation. And all the women were advised to undergo a regular biochemistry test (serum lipid was included) at the same time. In addition, early glucose screening (<24 weeks) is normally completed at the first prenatal visit in women with risk factors that include obesity with a BMI of ≥30 kg/m2, history of gestational diabetes in a prior pregnancy, known impaired glucose metabolism, hemoglobin A1C of ≥5.6%, first-degree relative with diabetes mellitus, high-risk ethnicity, history of polycystic ovarian syndrome, pre-existing hypertension or cardiovascular disease, or a prior large baby ≥4000 g. Thus, the data were collected at 20–28 gestational weeks.

### Diagnosis of GDM

All pregnant women at Nanjing Drum Tower hospital should subject to GDM screening, following an overnight fasting for 8–14 h, universally between 24 and 28 weeks’ gestation. According to the International Association of Diabetes and Pregnancy Study Groups criteria, the diagnosis of GDM was based on a 75 g OGTT (oral glucose tolerance test). The normal values were fasting plasma glucose < 5.1 mmol/L, 1-h glucose < 10.0 mmol/L and 2-h glucose < 8.5 mmol/L. GDM was diagnosed if one or more values equaled or exceeded the above thresholds [16].

### Diagnosis of hyperlipidemia

There is no consensus on which criteria should be used in diagnosis of hyperlipidemia. We combined the diagnostic criteria of dyslipidemia in general Chinese population (2016 revised edition) and clinical data, the diagnostic criteria for hyperlipidemia of second and third trimester was established: ①TC > 6.20 mmol/L and/or ②TG > 2.30 mmol/L.

### Statistical analysis

To ensure the normal distribution of variables, Histogram and Kolmogrov–Smirnov test were applied. Differences between groups were compared using the independent two sample t test and chi-squared test. Logistic regression analysis was performed to identify risk factors that were significantly correlated with GDM. Confounders were identified based on clinical experience and results of univariate analysis. The relevant confounding variables controlled for the multiple regression analysis included age, pre-pregnancy BMI, parity, gravidity, and gestational age at blood collection, and 95% CIs were calculated. All statistical analyses were performed using SPSS statistical software (Chicago, IL, USA). A *p* value < 0.05 was considered as statistically significant.

## Results

A total of 632 pregnant women were included in analyses. Of all of women, 359 were GDM and the remaining 273 were regarded as controls. Table 1 shows the general characteristics of the study group. Significant difference was observed in age between the two groups (29.30 ± 3.45 vs. 30.69 ± 4.20 years, *P* <  0.001). The pre-pregnancy BMI was significantly higher in the GDM group than that in the control group (22.86 ± 1.97 vs. 23.52 ± 2.42 kg/m^2^, *P* <  0.001). There was no significant difference between the two studied groups in terms of gestational weeks, gravidity, parity, residence, monthly income and education attainment (Table 1).

**Table 1:** Characteristics of the study population (means ± SD, n %)

	Control group (*n* = 273)	GDM group (*n* = 359)	*P*
Age (years)	29.30 ± 3.45	30.69 ± 4.20	< 0.001
Gestational age at blood collection (weeks)	22.01 ± 2.08	21.71 ± 2.03	0.234
pre-pregnancy BMI (kg/m^2^)	22.86 ± 1.97	23.52 ± 2.42	< 0.001
Gravidity (n, %)			0.491
1	120 (44.0)	148 (41.2)	
≥ 2	153 (56.0)	211 (58.8)	
Parity (n, %)			0.713
≤ 1	178 (65.2)	229 (63.8)	
≥ 2	95 (34.8)	130 (36.2)	
Residence (n, %)			0.093
Urban	240 (87.9)	330 (91.9)	
Rural area	33 (12.1)	29 (8.1)	
Monthly income (n, %)			0.752
≤ 6000	88 (32.2)	120 (33.4)	
>6000	185 (67.8)	239 (66.6)	
Education attainment (n, %)			0.725
≤ Secondary	130 (47.6)	176 (49.0)	
≥ College	143 (52.4)	183 (51.0)	

*BMI* Body mass index

Maternal lipid profile in the second trimester can be found in Table 2. Overall, women with GDM had higher levels of TG (2.05 ± 0.97 vs. 2.38 ± 1.37 mmol/L, *P* = 0.001), AIP (− 0.02 ± 0.20 vs. 0.08 ± 0.24, *P* <  0.001) and lower levels of HDL-C (2.02 ± 0.41 vs. 1.86 ± 0.42 mmol/L, *P* <  0.001) than that of control. TC, LDL-C, Apo A1 and Apo B were not significantly different between the groups. Regarding the pregnancy outcomes, there was more excessive postpartum hemorrhage (397.27 ± 213.61 vs. 446.17 ± 264.82 mL, *P* = 0.012) and higher rate of cesarean section (23.4 vs. 36.5%, *P* <  0.001) in GDM group than that in control group. The women in control group had larger gestational weeks at birth than the women in the GDM group (39.67 ± 1.38 vs. 38.79 ± 1.29 weeks, *P* <  0.001). As for the conditions of newborn, the birth weight was even lighter in the GDM group (3445.95 ± 519.96 vs. 3362.63 ± 443.63 g, *P* = 0.030). Body length of the newborn in GDM was slightly shorter compared with the control group (50.43 ± 1.75 vs. 50.08 ± 1.91 cm, *P* = 0.016). In addition, women with GDM had a higher rate of prematurity (1.8 vs. 7.3%, *P* = 0.002) while a lower rate of macrosomia (13.8 vs. 6.7%, *P* = 0.009). We failed to find significant differences in mean changes of Apgar score (5 min) between the two groups (*P* > 0.05).

**Table 2:** The levels of lipid indicators and pregnancy outcomes in GDM and non-GDM groups (means ± SD, n %)

	Control group (*n =* 273)	GDM group (*n =* 359)	*P*
TG (mmol/L)	2.05 ± 0.97	2.38 ± 1.37	0.001
TC (mmol/L)	5.56 ± 0.92	5.42 ± 0.92	0.050
HDL-C (mmol/L)	2.02 ± 0.41	1.86 ± 0.42	< 0.001
LDL-C (mmol/L)	2.69 ± 0.75	2.61 ± 0.73	0.159
Apo A1 (g/L)	2.42 ± 0.41	2.37 ± 0.41	0.195
Apo B (g/L)	1.06 ± 0.31	1.04 ± 0.22	0.486
AIP	−0.02 ± 0.20	0.08 ± 0.24	< 0.001
Cesarean section (n, %)	64 (23.4)	131 (36.5)	< 0.001
Postpartum hemorrhage (ml)	397.27 ± 213.61	446.71 ± 264.82	0.012
Gestational weeks at birth (weeks)	39.67 ± 1.38	38.79 ± 1.29	< 0.001
Birth weight (g)	3445.95 ± 519.96	3362.63 ± 443.63	0.030
Body length (cm)	50.43 ± 1.75	50.08 ± 1.91	0.016
Prematurity (n, %)	5 (1.8)	19 (7.3)	0.002
Macrosomia (n, %)	35 (13.8)	24 (6.7)	0.009
Apgar 5 min score	9.98 ± 0.17	9.98 ± 0.17	0.982

*AIP* Atherogenic index of plasma, *Apo A1/B* Apolipoprotein A1/B, *BMI* Body mass index, *GDM* Gestational diabetes mellitus, *HDL-C* High-density lipoprotein-cholesterol, *LDL-C* Low-density lipoprotein-cholesterol, *SD* Standard deviation, *TC* Total cholesterol, *TG* Triglycerides

Maternal hyperlipidemia was defined as ①TC > 6.20 mmol/L or ②TG > 2.30 mmol/L (Table 3). When grouped by lipid concentrations, the incidence of GDM (47.3 vs. 64.6%, *P* <  0.001) and cesarean section (27.3 vs. 34.8%, *P* = 0.042) was significantly increased in the hyperlipidemia group. Compared with women in the normal lipid group, those in the hyperlipidemia group had smaller gestational weeks at birth (39.32 ± 1.248 vs. 39.00 ± 1.54 weeks, *P* = 0.005). No statistically significant difference was seen between the two groups in terms of pre-pregnancy BMI, postpartum hemorrhage, birth weight, the rate of prematurity, macrosomia,and Apgar score (5 min) (*P* > 0.05).

**Table 3:** Clinical data grouped by lipid concentrations (means ± SD, n %)

	Normal lipid (*n* = 330)	Hyperlipidemia (*n* = 302)	*P*-value
GDM (n,%)	164 (47.3)	195 (64.6)	< 0.001
pre-pregnancy BMI (kg/m^2^)	23.16 ± 2.21	23.31 ± 2.31	0.422
Cesarean section (n,%)	90 (27.3)	105 (34.8)	0.042
Postpartum hemorrhage (n,%)	96 (29.1)	77 (25.5)	0.311
Gestational weeks at birth (weeks)	39.32 ± 1.24	39.00 ± 1.54	0.005
Birth weight (g)	3388.66 ± 449.41	3424.54 ± 482.19	0.333
Prematurity (n,%)	13 (3.9)	11 (3.6)	0.845
Macrosomia (n, %)	25 (7.6)	34 (11.3)	0.112
Apgar 5 min score	9.98 ± 0.13	9.97 ± 0.19	0.530

*BMI* Body mass index, *GDM* Gestational diabetes mellitus, *SD* Standard deviation

We then investigated whether there was an association between maternal serum lipids and the risk of GDM. After adjusting for age, pre-pregnancy BMI, parity, gravidity and gestational age at blood collection, the risk of GDM increased approximately 18.48-fold with each unit elevation in AIP (OR = 18.48, 95%CI: 2.38 ~ 143.22). Meanwhile, GDM had a positive correlation with age (OR = 1.11, 95%CI: 1.06 ~ 1.16) and pre-pregnancy BMI (OR = 1.15, 95% CI: 1.07 ~ 1.24). Other lipid variables were almost no contribution to the risk of GDM (Table 4).

**Table 4:** Logistic regression analysis of GDM and lipid levels

Factors	GDM			
	*B*	*P*	OR	95% CI
TG (mmol/L)	−0.002	0.993	1.00	0.62 ~ 1.61
TC (mmol/L)	−0.397	0.058	0.67	0.45 ~ 1.01
HDL (mmol/L)	0.054	0.511	1.06	0.900 ~ 1.24
LDL (mmol/L)	0.374	0.198	1.45	0.82 ~ 2.57
Apo A1 (g/L)	0.363	0.208	1.44	0.82 ~ 2.53
Apo B (g/L)	−0.441	0.475	0.64	0.19 ~ 2.16
AIP	2.917	0.005	18.48	2.38 ~ 143.22
Age (years)	0.102	<0.001	1.11	1.06 ~ 1.16
Gestational age at blood collection (weeks)	−0.049	0.272	0.95	0.87 ~ 1.04
Pre-pregnancy BMI (kg/m^2^)	0.141	<0.001	1.15	1.07 ~ 1.24
Gravidity (n)	0.148	0.547	1.16	0.72 ~ 1.87
Parity (n)	0.018	0.944	1.02	0.62 ~ 1.66

*AIP* Atherogenic index of plasma, *Apo A1/B* Apolipoprotein A1/B, *BMI* Body mass index, *GDM* Gestational diabetes mellitus, *HDL-C* High-density lipoprotein-cholesterol, *LDL-C* Low-density lipoprotein-cholesterol, *SD* Standard deviation, *TC* Total cholesterol, *TG* Triglycerides

## Discussion

Maternal lipid metabolism changes apparently throughout gestation. During the 1st two-thirds of gestation, there is an elevation in fat accumulation, related to hyperphagia and increased lipogenesis [17, 18]. In the last 3rd trimester of gestation, maternal fat storage decreases due to the enhanced lipolytic activity and declined lipoprotein lipase activity [19, 20]. These alterations are reflections of maternal physiologic adaptation to energy demand of the fetus, as well as preparations for delivery and lactation [21].

GDM is strongly associated with an increased risk of short-term and long-term complications for both mothers and offspring [6–8]. In this study, postpartum hemorrhage in the GDM group was greater than that in the control group. In addition, patients with GDM had an increased rate of prematurity, as well as a lower incidence of macrosomia. Although it is well known that diabetic pregnancies are associated with a high incidence of fetal growth disorders/macrosomia, it is now evident that control of fetal growth is far more complex than previously thought [22]. Evidence also suggested that increases in maternal plasma triacylglycerols (TAGs) levels in GDM women were related to neonates that were either large or small for their gestational age (LGA or SGA )[23, 24]. Previous studies demonstrated a poor rate of weight gain during the latter part of pregnancy was considered to be associated with an increased risk of SGA and preterm delivery [25, 26]. In clinical practice, we have observed that patients with GDM had a better understanding of food choice and pay more attention to weight control. We then speculated that, compared to health pregnancies, women with GDM had a relative lower rate of weight gain during pregnancy. Further robust clinical trails are warranted to corroborate this observation.

Most studies showed that circulating lipid patterns were different between GDM and normal pregnancy, nevertheless, results have been inconsistent [27]. It has been reported that patients with GDM had increased concentrations of TG, TC and LDL-C and lower levels of HDL-C [28, 29]. However, other studies indicated that no elevated serum TC and LDL-C levels were found in the 1st, 2nd, and 3rd trimesters, between patients with GDM and normal pregnant women [30]. In present study, we found that women with GDM had higher levels of TG and lower levels of HDL-C than that of control. AIP is a good predictor of the risk of atherosclerosis and coronary heart disease. Previous research found that diabetic patients had statistically significantly higher levels of AIP than the control group [31–33]. In concordance with these reports, our results also showed that the level of AIP among women with GDM was higher than the non-GDM population. Thus, our data suggested abnormal maternal lipids have a role in the pathogenesis of GDM.

Although changes in lipid profile are expected, it is still difficult to determine the cut-off level for diagnosing hyperlipidemia in pregnant women. We combined the diagnostic criteria of dyslipidemia in general Chinese population and clinical data during pregnancy, pregnant women whose TC > 6.20 mmol/L and TG > 2.30 mmol/L or to either of them were diagnosed with hyperlipidemia. Expect for pregnancy complications [15], maternal dyslipidemia has also been proved to be closely linked with adverse pregnancy outcomes [14]. We found that women with hyperlipidemia compared with these in normal lipid group had higher incidence of GDM and cesarean section. The exact definition of hyperlipidemia and its effect on the risk of GDM are not clearly understood [11, 30]. We then to explore whether dyslipidemia in the 2nd trimester has potential clinical utility for identifying women at risk for developing GDM. Logistic regression analysis showed that AIP, age and pre-pregnancy BMI were positively correlated with GDM, and AIP was a risk factor of GDM. Collectively, recognizing lipid abnormalities may allow for appropriate risk-reducing interventions of GDM.

As for the conditions of newborn, current evidence shows conflicting results about the relationship between maternal dyslipidemia and adverse neonatal outcomes. Recently, some maternal lipid parameters have been served as independent predictors of fetal overgrowth, especially in women complicated by GDM [34, 35]. The risk of macrosomia was positively related to TG levels, while negatively related to HDL-C levels in non-diabetic pregnancies [36]. However, other studies failed to find any association [37, 38]. In the present study, we didn’t find significant differences in the incidence of prematurity and macrosomia between the normal lipid and hyperlipidemia groups. The inconsistency may be due to the different study populations and sample size. Collectively, there is a need to improve our knowledge about the maternal lipid profile change in relation to fetal growth.

### Study strength and limitations

This study helps to establish the standard of hyperlipidemia diagnosis during pregnancy in Chinese population. This study also had several limitations. First, confounding factors were the main limitations of this retrospective observational design. For example, the information regarding the body weight during pregnancy and lifestyles (eg. dietary factors) was missing. Second, participants recruited in the present study may not represent the general population of pregnant woman due to the selection bias and sample size, which might influence on the analysis of the relationship between mid-trimester lipid profile and GDM. Third, a relatively short duration of observation. Lipid concentrations could change constantly through gestational weeks. Finally, it would be better to perform a longitudinal study to show the association between serum lipids and GDM.

## Conclusions

In conclusion, this study added to the current knowledge of associations between changes occurred in lipid metabolism during pregnancy and outcomes for mothers and newborns in women with GDM. Further studies are also warranted to establish the standard of hyperlipidemia diagnosis during pregnancy according to local maternal characteristics. More attention should be paid to the mid-trimester lipid profile in all mothers in clinical practice. As for pregnant woman with abnormal blood lipids, close monitoring and lifestyle management during pregnancy should be carried out to prevent pregnancy complications and improve clinical outcomes as much as possible.

## Data Availability

The datasets used and/or analysed during the current study are available from the corresponding author on reasonable request.

## Abbreviations

AIP: Atherogenic index of plasma
Apo A1/B: Apolipoprotein A1/B
BMI: Body mass index
GDM: Gestational diabetes mellitus
GWG: Gestational weight gain
HDL-C: High-density lipoprotein-cholesterol
LDL-C: Low-density lipoprotein-cholesterol
OR: Odds ratio
SGA: Small for gestational age
SD: Standard deviation
TC: Total cholesterol
TG: Triglycerides
TAGs: Triacylglycerols

## References

[1] Nasioudis D, Doulaveris G, Kanninen TT (2019). Dyslipidemia in pregnancy and maternal-fetal outcome. Minerva Ginecol.

[2] Herrera E, Desoye G (2016). Maternal and fetal lipid metabolism under normal and gestational diabetic conditions. Horm Mol Biol Clin Invest.

[3] Brizzi P, Tonolo G, Esposito F, Puddu L, Dessole S, Maioli M (1999). Lipoprotein metabolism during normal pregnancy. Am J Obstet Gynecol.

[4] Lippi G, Albiero A, Montagnana M, Salvagno GL, Scevarolli S, Franchi M (2007). Lipid and lipoprotein profile in physiological pregnancy. Clin Lab.

[5] Ryckman KK, Spracklen CN, Smith CJ, Robinson JG, Saftlas AF (2015). Maternal lipid levels during pregnancy and gestational diabetes: a systematic review and meta-analysis. BJOG..

[6] Sacks DA, Black MH, Li X, Montoro MN, Lawrence JM (2015). Adverse pregnancy outcomes using the International Association of the Diabetes and Pregnancy Study Groups Criteria: glycemic thresholds and associated risks. Obstet Gynecol.

[7] Sreelakshmi PR, Nair S, Soman B, Alex R, Vijayakumar K, Kutty VR (2015). Maternal and neonatal outcomes of gestational diabetes: a retrospective cohort study from southern India. J Family Med Prim Care.

[8] Muche AA, Olayemi OO, Gete YK (2020). Effects of gestational diabetes mellitus on risk of adverse maternal outcomes: a prospective cohort study in Northwest Ethiopia. BMC Pregnancy Childbirth.

[9] Herrera Martinez A, Palomares Ortega R, Bahamondes Opazo R, Moreno-Moreno P (2018). Molina Puerta mf, Galvez-Moreno MA. Hyperlipidemia during gestational diabetes and its relation with maternal and offspring complications. Nutr Hosp.

[10] Emet T, Ustuner I, Guven SG, Balik G, Ural UM, Tekin YB (2013). Plasma lipids and lipoproteins during pregnancy and related pregnancy outcomes. Arch Gynecol Obstet.

[11] Shen H, Liu X, Chen Y, He B, Cheng W (2016). Associations of lipid levels during gestation with hypertensive disorders of pregnancy and gestational diabetes mellitus: a prospective longitudinal cohort study. BMJ Open.

[12] Mudd LM, Holzman CB, Catov JM, Senagore PK, Evans RW (2012). Maternal lipids at mid-pregnancy and the risk of preterm delivery. Acta Obstet Gynecol Scand.

[13] Wang J, Moore D, Subramanian A, Cheng KK, Toulis KA, Qiu X (2018). Gestational dyslipidaemia and adverse birthweight outcomes: a systematic review and meta-analysis. Obes Rev.

[14] Mohsenzadeh-Ledari F, Taghizadeh Z, Motaghi Z, Keramat A, Moosazadeh M, Najafi A (2019). Appropriate interventions for pregnant women with indicators of metabolic syndrome on pregnancy outcomes: a systematic review. Int J Prev Med.

[15] Jin WY, Lin SL, Hou RL, Chen XY, Han T, Jin Y (2016). Associations between maternal lipid profile and pregnancy complications and perinatal outcomes: a population-based study from China. BMC Pregnancy Childbirth.

[16] Weinert LS (2010). International Association of Diabetes and Pregnancy Study Groups recommendations on the diagnosis and classification of hyperglycemia in pregnancy: comment to the International Association of Diabetes and Pregnancy Study Groups Consensus Panel. Diabetes Care.

[17] Murphy SP, Abrams BF (1993). Changes in energy intakes during pregnancy and lactation in a national sample of US women. Am J Public Health.

[18] Mankuta D, Elami-Suzin M, Elhayani A, Vinker S (2010). Lipid profile in consecutive pregnancies. Lipids Health Dis.

[19] Herrera E, Lasuncion MA, Gomez-Coronado D, Aranda P, Lopez-Luna P, Maier I (1988). Role of lipoprotein lipase activity on lipoprotein metabolism and the fate of circulating triglycerides in pregnancy. Am J Obstet Gynecol.

[20] Meyer BJ, Stewart FM, Brown EA, Cooney J, Nilsson S (2013). Olivecrona get al. Maternal obesity is associated with the formation of small dense LDL and hypoadiponectinemia in the third trimester. J Clin Endocrinol Metab.

[21] Di Cianni G, Miccoli R, Volpe L, Lencioni C, Ghio A, Giovannitti MG (2005). Maternal triglyceride levels and newborn weight in pregnant women with normal glucose tolerance. Diabet Med.

[22] Higgins M, Mc AF (2010). A review of maternal and fetal growth factors in diabetic pregnancy. Curr Diabetes Rev.

[23] Simeonova-Krstevska S, Krstevska B, Velkoska-Nakova V, Hadji Lega M, Samardjiski I, Serafimoski V (2014). Effect of lipid parameters on foetal growth in gestational diabetes mellitus pregnancies. Pril (Makedon Akad Nauk Umet Odd Med Nauki).

[24] Kitajima M, Oka S, Yasuhi I, Fukuda M, Rii Y, Ishimaru T (2001). Maternal serum triglyceride at 24--32 weeks' gestation and newborn weight in nondiabetic women with positive diabetic screens. Obstet Gynecol.

[25] Kashyap H, Sharma D, Gala A, Pratap OT, Murki S (2019). Effect of second trimester and third trimester weight gain on immediate outcomes in neonates born to mothers with gestational diabetes: a retrospective observational study from India. J Matern Fetal Neonatal Med.

[26] Carmichael SL, Abrams B (1997). A critical review of the relationship between gestational weight gain and preterm delivery. Obstet Gynecol.

[27] Huang A, Ji Z, Zhao W, Hu H, Yang Q, Chen D (2016). Rate of gestational weight gain and preterm birth in relation to prepregnancy body mass indices and trimester: a follow-up study in China. Reprod Health.

[28] Savvidou M, Nelson SM, Makgoba M, Messow CM, Sattar N, Nicolaides K (2010). First-trimester prediction of gestational diabetes mellitus: examining the potential of combining maternal characteristics and laboratory measures. Diabetes..

[29] Li G, Kong L, Zhang L, Fan L, Su Y, Rose JC (2015). Early pregnancy maternal lipid profiles and the risk of gestational diabetes mellitus stratified for body mass index. Reprod Sci.

[30] Wang J, Li Z, Lin L (2019). Maternal lipid profiles in women with and without gestational diabetes mellitus. Medicine (Baltimore).

[31] Fadel MM, Abdel Ghaffar FR, Zwain SK, Ibrahim HM, Badr EA (2021). Serum netrin and VCAM-1 as biomarker for Egyptian patients with type IIota diabetes mellitus. Biochem Biophys Rep.

[32] Gasevic D, Frohlich J, Mancini GB, Lear SA (2012). The association between triglyceride to high-density-lipoprotein cholesterol ratio and insulin resistance in a multiethnic primary prevention cohort. Metabolism..

[33] Khosrowbeygi A, Shiamizadeh N, Taghizadeh N (2016). Maternal circulating levels of some metabolic syndrome biomarkers in gestational diabetes mellitus. Endocrine..

[34] Schaefer-Graf UM, Graf K, Kulbacka I, Kjos SL, Dudenhausen J, Vetter K (2008). Maternal lipids as strong determinants of fetal environment and growth in pregnancies with gestational diabetes mellitus. Diabetes Care.

[35] Son GH, Kwon JY, Kim YH, Park YW (2010). Maternal serum triglycerides as predictive factors for large-for-gestational age newborns in women with gestational diabetes mellitus. Acta Obstet Gynecol Scand.

[36] Wang X, Guan Q, Zhao J, Yang F, Yuan Z, Yin Y (2018). Association of maternal serum lipids at late gestation with the risk of neonatal macrosomia in women without diabetes mellitus. Lipids Health Dis.

[37] Retnakaran R, Ye C, Hanley AJ, Connelly PW, Sermer M, Zinman B (2012). Effect of maternal weight, adipokines, glucose intolerance and lipids on infant birth weight among women without gestational diabetes mellitus. CMAJ..

[38] Parlakgumus HA, Aytac PC, Kalayci H, Tarim E (2014). First trimester maternal lipid levels and serum markers of small- and large-for-gestational age infants. J Matern Fetal Neonatal Med.

